# Genome-Wide Identification, Characterization, and Expression Analysis of *BES1* Family Genes in ‘*Tieguanyin*’ Tea Under Abiotic Stress

**DOI:** 10.3390/plants14030473

**Published:** 2025-02-05

**Authors:** Yanzi Zhang, Yanlin Zhang, Zhicheng Yang, Qingyan Li, Weixiang Chen, Xinyan Wen, Hao Chen, Shijiang Cao

**Affiliations:** 1 Metabolomics Center, Haixia Institute of Science and Technology, Fujian Agriculture and Forestry University, Fuzhou 350002, China; zhangyanzi163@163.com; 2 College of Jun Cao Science and Ecology (College of Carbon Neutrality), Fujian Agriculture and Forestry University, Fuzhou 350002, China; 17770899282@163.com (Y.Z.); 15255665669@163.com (Q.L.); 3 College of Future Technologies, Fujian Agriculture and Forestry University, Fuzhou 350002, China; 18359797810@163.com (Z.Y.); 19896657097@163.com (W.C.); 4 College of Food Science, Fujian Agriculture and Forestry University, Fuzhou 350002, China; dalethandalef@outlook.com; 5 College of Computer and Information Sciences, Fujian Agriculture and Forestry University, Fuzhou 350002, China; 6 College of Forestry, Fujian Agriculture and Forestry University, Fuzhou 350002, China

**Keywords:** *Tieguanyin*, *BES1*, gene family, abiotic stress, expression analysis

## Abstract

The *BRI1-EMS-SUPPRESSOR 1* (*BES1*) family comprises plant-specific transcription factors, which are distinguished by atypical bHLH domains. Over the past two decades, genetic and biochemical studies have established that members of the *BRI1-EMS-SUPPRESSOR 1* (*BES1*) family are crucial for regulating the expression of genes involved in brassinosteroid (BR) response in rapeseed. Due to the significance of the *BES1* gene family, extensive research has been conducted to investigate its functional properties. This study presents a comprehensive identification and computational analysis of *BES1* genes in ‘*Tieguanyin*’ (*TGY*) tea (*Camellia sinensis*). A total of 10 *BES1* genes were initially identified in the *TGY* genome. Through phylogenetic tree analysis, this study uniquely revealed that *CsBES1.2* and *CsBES1.5* cluster with *SlBES1.8* from *Solanum lycopersicum*, indicating their critical roles in fruit growth and development. Synteny analysis identified 20 syntenic genes, suggesting the conservation of their evolutionary functions. Analysis of the promoter regions revealed two types of light-responsive cis-elements, with *CsBES1.4* exhibiting the highest number of light-related cis-elements (13), followed by *CsBES1.9* and *CsBES1.10*. Additional validation via qRT-PCR experiments showed that *CsBES1.9* and *CsBES1.10* were significantly upregulated under light exposure, with *CsBES1.10* reaching approximately six times the expression level of the control after 4 h. These results suggest that *CsBES1.9* and *CsBES1.4* could play crucial roles in responding to abiotic stress. This study offers novel insights into the functional roles of the *BES1* gene family in ‘*Tieguanyin*’ tea and establishes a significant foundation for future research, especially in exploring the roles of these genes in response to abiotic stresses, such as light exposure.

## 1. Introduction

**Figure 1:**
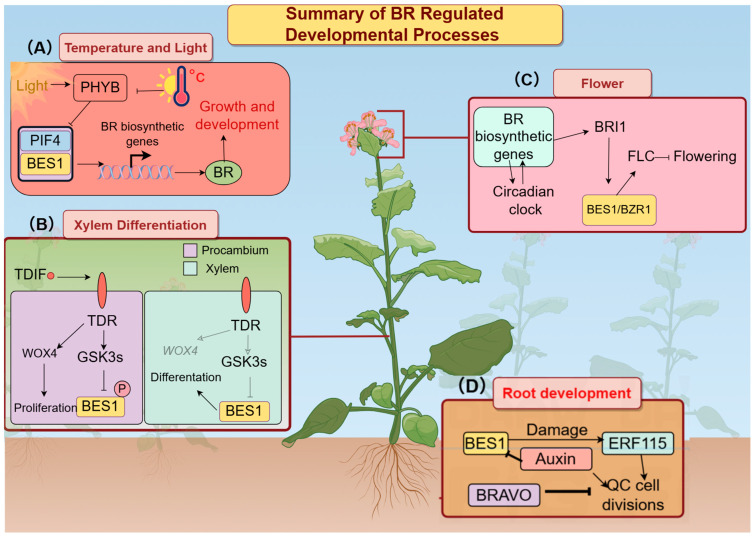
Summary of BR regulating developmental processes in *A. thaliana*. (**A**) High temperature inhibits thTFs, determines their gene targets, and leads to different cellular responses. (**B**) Xylem differentiation is controlled by the TRACHEARY ELEMENT DIFFERENTIATION INHIBITORY FACTOR (TDIF) signaling pathway (the red dots represent the TDIF signal). GSK3 is a key component in this pathway. It acts as a negative regulator of xylem differentiation and crosstalks with the brassinosteroid (BR) signaling pathway. The TDIF signal acts on the TDR receptor on cambium cells, resulting in the inhibition of *BRS1* activity by GSK3s. In the xylem where it is not inhibited by the activity of PHYB, light promotes the activity of PHYB. PHYB inhibits the production of PIF4 and indirectly determines the level of PIF4-BES1 heterodimerization. The interactions among these transcription factors (DIF and *BES1*) functions to promote cell elongation (the gray arrows indicate that no has reaction occurred). (**C**) Brassinosteroids (BRs) inhibit flowering by promoting the expression of the flowering inhibitor FLC. (**D**) In the root apical meristem, BRs control the size of the stem cell niche by balancing the expression of BRAVO, which negatively regulates cell division in the quiescent center. BR signaling levels increase along the longitudinal axis, with higher levels present in cells closer to the differentiation/elongation zone. Arrows indicate activation and blunt-ended lines indicate inhibition.

Brassinosteroids (BRs), a group of steroidal plant hormones, play a crucial role in regulating plant growth and numerous physiological functions. Maintaining appropriate BR levels is essential for proper root development and the overall growth of plants [1,2,3,4,5,6,7]. Brassinosteroids (BRs) were the sixth class of plant hormones to be discovered, following auxins, gibberellins, cytokinins, abscisic acid, and ethylene [8,9]. They promote cell elongation by stimulating hormone accumulation in meristematic tissues, thereby transitioning cells into elongation zones. In *Arabidopsis thaliana*, mutants deficient in brassinosteroids (BRs) exhibit traits such as dwarfism, impaired cell expansion, reduced apical dominance, delayed flowering and senescence, and male sterility [10,11]. Moreover, the BR signaling pathway interacts with various response networks to coordinate plant growth, development, and adaptation to abiotic stresses [8]. For details, please refer to Figure 1.

For instance, BRs negatively regulate the plant drought stress response via RESPONSIVE TO DESICCATION 26 (RD26) and interact with light, abscisic acid (ABA), and gibberellic acid (GA) signaling pathways through *BES1* [2,12,13,14].

Transcription factors (TFs) regulate the expression of specific target genes in response to biotic and abiotic stresses, playing key roles in plant growth, development, and stress resistance. Through the activation of various defense mechanisms, they influence yield and quality [15,16]. *BRI1-EMS-SUPPRESSOR 1* (*BES1*) and *BRASSINAZOLE RESISTANT 1* (*BZR1*) are key transcription factors that directly mediate BR signaling [2,17,18,19,20]. *BES1* and *BZR1* exhibit significant sequence similarity and serve as crucial regulators in plant responses to stress [3,17,21,22,23,24,25]. Both function as activators of the BR signaling pathway [26]. Studies have shown that a conserved DNA-binding motif at the N-terminus of *BES1*-type proteins binds to E-box or BR response elements, thereby regulating the transcription of target genes [2,3,19,21]. In plant cells, when a brassinosteroid (BR) binds to the extracellular domain of the BR receptor BRI1, it activates the intracellular kinase activity of the receptor.

This activation triggers the release of BKI1 from the cell membrane and facilitates the interaction between BRI1 and BAK1, thereby transmitting BR signals into the cell [27,28]. In the cytoplasm, *BR SIGNAL KINASE 1* (*BSK1*) activates *BRI1 SUPPRESSOR 1* (*BSU1*) via a phosphorylation-dephosphorylation cascade. This process leads to the dephosphorylation and degradation of *BR-INSENSITIVE 2* (*BIN2*), thus alleviating the inhibition of *BES1* [29,30,31]. Additionally, *BES1* undergoes dephosphorylation by *PROTEIN PHOSPHATASE 2A* (*PP2A*), which facilitates its accumulation in the nucleus and regulates downstream target genes [32,33]. In addition to mediating BR signaling, *BES1*/*BZR1* integrates multiple plant hormone pathways, controlling tapetum development and various stress responses [34,35,36,37,38,39,40,41]. *BES1* interacts with TFs such as phytochrome interacting factor 4 (PIF4), WRKY46, WRKY54, and WRKY70 to regulate cell expansion and enhance stress resistance [42,43].

*A. thaliana* contains eight members of the *BES1* family, whereas other species, such as rapeseed (*Brassica napus*), cotton (*Gossypium hirsutum*), maize (*Zea mays*), tomato (*Solanum lycopersicum*), cabbage (*Brassica oleracea*), and cucumber (*Cucumis sativus*), possess varying numbers of *BES1* homologs: 28, 22, 11, 9, 15, and 6, respectively [34,44,45,46,47,48,49]. *BES1*s are involved in cell growth [50], pollen development [51], plant immunity [52], and stress responses. For example, in cotton, the *GhBES1* gene displays functional variability, influencing fiber development, plant morphology, and stress resistance [46]. The cotton variety ‘*Xinluzao 17*’ shows a rapid response to drought stress, which is regulated by *BES1*. In tomatoes, *BES1* expression is significantly upregulated following salt stress at 6 and 24 h [47,53].

The tea plant, *Camellia sinensis* (L.) O. Kuntze, is native to southwestern China, where it has been grown and used for thousands of years [54]. Tea, the second most consumed beverage globally, is rich in amino acids, vitamins, minerals, tea polyphenols, and alkaloids [55]. It is renowned for its health benefits, including its antioxidant, anti-tumor, and cardiovascular properties [56,57,58]. Environmental conditions significantly affect the growth of tea plants, with factors such as extreme temperatures, drought, and light stress playing critical roles in their natural habitats [59]. Among these factors, light is a crucial environmental factor influencing various economic traits, including leaf size, shoot weight, harvest duration, and the synthesis of health-promoting compounds [60,61,62]. Previous research has demonstrated that *BES1*/*BZR1* is essential for regulating light signaling in plant development [63].

Its phosphorylation status and stability are regulated by light signals, with dephosphorylation enhancing activity in the dark and phosphorylation by BIN2 keeping it in an inactive state under light [64]. The increasing frequency of extreme weather events, including high temperatures and droughts, exacerbated by global climate change, has caused significant damage to tea production. To improve both the yield and quality of tea, understanding the mechanisms underlying plants’ tolerance to environmental stress is essential. Therefore, investigating the molecular mechanisms by which *BES1* responds to light and dark stress is vital for optimizing the growth and development of tea plants.

Among oolong teas, ‘*Tieguanyin*’ (*TGY*) is notable for its high concentration of nutritional and medicinal compounds. However, research on the *BES1* gene family in *TGY* is still limited. Few studies have investigated changes in *BES1* expression under light stress, despite its growing significance in the context of environmental challenges. This study systematically identified the *BES1* gene family in *TGY* through bioinformatics, analyzing its physicochemical properties, evolutionary relationships, gene architecture, conserved domains, and intraspecies/interspecies variation. The expression profile of the *CsBES1* gene was analyzed to provide insights into its role in adapting *TGY* tea to environmental stresses, improving quality, increasing yield, and developing stress-resistant crops in the face of global climate change.

## 2. Results

### 2.1. Identification and Characterization of CsBES1 Genes

The amino acid sequences of ten members of the *CsBES1* family were predicted and labeled as *CsBES1.1* to *CsBES1.10* (details provided in Table 1). The size of the protein is generally positively correlated with the length of the amino acid sequence. Within the *CsBES1* family, the number of amino acids varies significantly, ranging from 315 in *CsBES1.5* to 699 in *CsBES1.6*. The molecular weight of these proteins ranges from 34,258.36 kDa (*CsBES1.9*) to 78,007.57 kDa (*CsBES1.6*), while their theoretical isoelectric points (pI) range from 5.37 (*CsBES1.6*) to 10.02 (*CsBES1.1*), with an average of 8.17. Variations in pI suggest that *CsBES1* proteins function in diverse microenvironments.

All ten *CsBES1* proteins exhibited negative hydrophilicity, classifying them as hydrophilic proteins. The lipid solubility indices of these proteins ranged from 37.02 (*CsBES1.5*) to 81.82 (*CsBES1.3*), with four proteins exhibiting indices above 70, suggesting heat stability. Proteins with instability coefficients below 40 are typically considered stable, whereas those with coefficients above 40 are classified as unstable. Among the ten *CsBES1* family members, only *CsBES1.5* and *CsBES1.6* had instability coefficients below 40, indicating that they are stable proteins. The remaining members were predicted to be unstable.

Subcellular localization analysis also revealed that approximately nine *CsBES1* proteins are localized in the nucleus. Notably, *CsBES1.5* was the only member predicted to be localized in the chloroplast, emphasizing potential functional diversity within the family.

### 2.2. The Motif, Domain, and Gene Structure of CsBES1 Family

**Figure 2:**
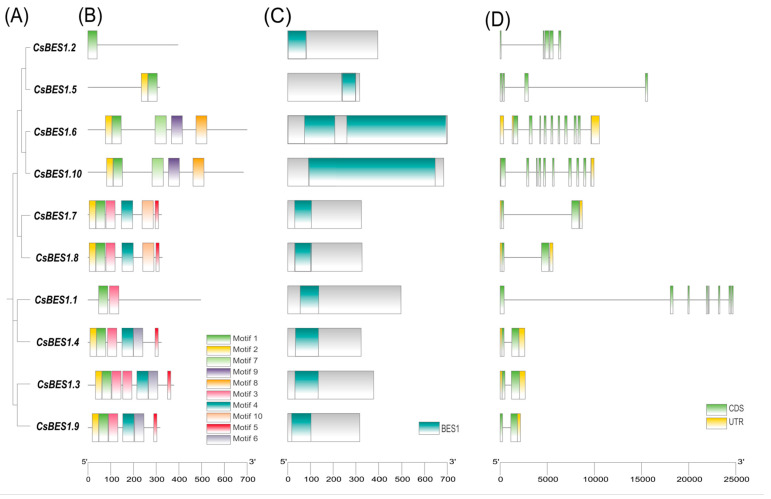
Protein motifs, domains, and structures of the *BES1* gene family in ‘*Tieguanyin*’. (**A**) A phylogenetic tree of *CsBES1* proteins was constructed using MEGAX 12.0 software. The tree was generated with the maximum likelihood method and validated through 1000 bootstrap replications, ensuring consistency and reliability. (**B**) Protein motifs in *CsBES1* members are represented by colorful boxes, each denoting a distinct motif. (**C**) Conserved domains are depicted, illustrating the shared structural features of the *BES1* proteins. (**D**) The gene structures of the *CsBES1* gene family are shown. Coding sequence (CDS) regions are represented by green rectangles, untranslated regions (UTRs) are represented by yellow rectangles, and introns are represented by black lines.

Gene structure is a key characteristic of gene families, offering valuable insights into their evolutionary history [65]. Conserved protein domains, characterized by high sequence similarity, provide essential insights into evolutionary relationships [66]. To explore the evolutionary relationships within the *BES1* family, 10 conserved protein motifs and their structures were analyzed. Using MEME, 10 conserved motifs were identified among the 10 *BES1* proteins (Figure 2). Most family members cluster within the same branch and share similar motif and domain structures, although notable diversity exists among *CsBES1* proteins. Nearly all *CsBES1* proteins contain motif 1, the most highly conserved motif across the family, emphasizing its potential functional significance. The widespread presence of motif 1 suggests a critical role in fundamental biological activities, possibly involving DNA binding or protein–protein interactions.

With the exception of *CsBES1.2* and *CsBES1.1*, all proteins contain motif 2, indicating its relatively conserved nature. Notably, *CsBES1.6* and *CsBES1.10* both possess motifs 1, 2, 7, 9, and 8, while *CsBES1.7* and *CsBES1.8* share motifs 1, 2, 3, 4, 10, and 5, suggesting potential functional similarities. These proteins may regulate similar sets of genes or biological processes. Additionally, conserved domains were identified across all *CsBES1* proteins, further supporting a high degree of conservation. This finding highlights the need for further exploration of their diverse biological functions.

Exon–intron structure analysis revealed a total of 10 genes. Notably, *CsBES1.2* and *CsBES1.5* lack non-coding regions, which are crucial for regulating mRNA stability and gene expression. Interestingly, *CsBES1* members within the same group exhibit minimal variation in exon and intron number and length, reinforcing the functional conservation of BES1 genes within these groups.

### 2.3. Phylogenetic Analysis of CsBES1s

**Figure 3:**
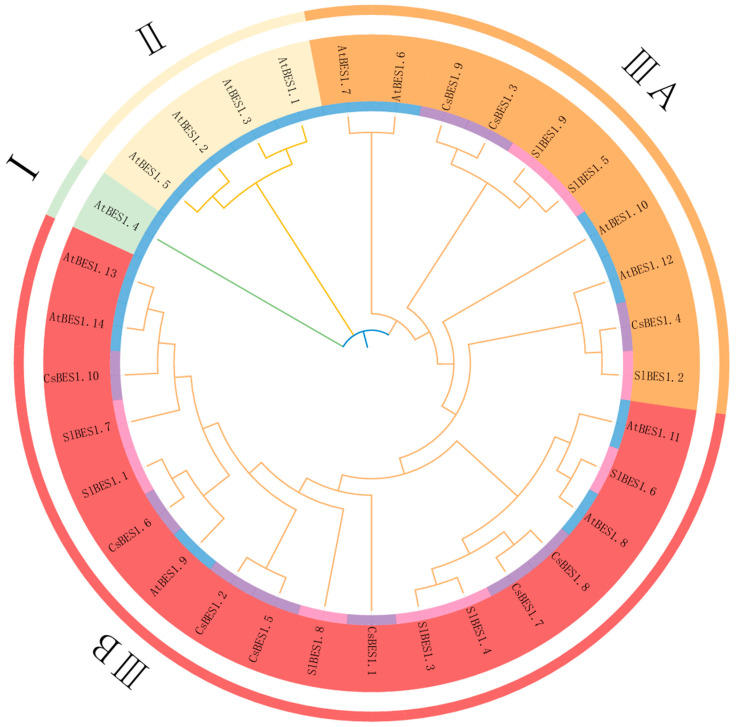
A phylogenetic analysis of *BES1* proteins from *Tieguanyin* (Cs), *A. thaliana* (At), and *Solanum lycopersicum* (Sl) was conducted using the neighbor-joining method with the maximum likelihood approach and 1000 bootstrap replicates. Different colors in the inner circle represent the three species, while the four subgroups of *BES1* proteins (Groups I–IIIB) are distinguished by varying colors in the outer circle.

The phylogenetic tree illustrates the evolutionary relationships among members of the *CsBES1* gene family and their counterparts in other species. To further explore these relationships, a multiple sequence alignment and a maximum likelihood phylogenetic tree were constructed, with *Arabidopsis* and *Solanum lycopersicum* as reference species. The *CsBES1* family in *TGY* comprises 10 members, while *A. thaliana* and *S. lycopersicum* have 14 and 9 members, respectively, yielding a total of 33 members, which are classified into four groups (I–IIIB) based on the grouping in *A. thaliana* (Figure 3). The distribution of *CsBES1* genes among these groups is uneven. Group IIIB contains the majority of *CsBES1* members (*CsBES1.1*, *CsBES1.2*, *CsBES1.5*, *CsBES1.6*, *CsBES1.7*, *CsBES1.8*, and *CsBES1.10*), whereas Group IIIA includes three members (*CsBES1.3*, *CsBES1.4*, and *CsBES1.9*). Groups I and II consist only of *BES1* genes from *A. thaliana*, suggesting that *BES1* in *TGY* underwent expansion after the divergence of monocots and retains conserved features.

Martin’s analysis suggests that *BES1* genes originated during the bryophyte stage of biological evolution and further evolved after the phytoplankton stage [67]. Within Group IIIB, *SlBES1.1* and *CsBES1.6*, as well as *SlBES1.7* and *CsBES1.10*, form direct homologous relationships. Similarly, *CsBES1.2* and *CsBES1.5* cluster with *SlBES1.8*. Notably, *SlBES1.8* plays a critical role in fruit setting and development in *S. lycopersicum*, with its activity being induced by auxin and gibberellin [19]. Since *CsBES1.1* is a direct homolog of *SlBES1.8*, it is plausible to hypothesize that *CsBES1.1* may play a similar role in *TGY* tea development. Further analysis revealed that *CsBES1.10* clusters with *AtBES1.13* and *AtBES1.14*, while *CsBES1.6* and *AtBES1.9* are orthologous pairs, potentially involved in similar regulatory pathways.

### 2.4. Chromosome Distribution of CsBES1s and Genomic Amplification in TGY

**Figure 4:**
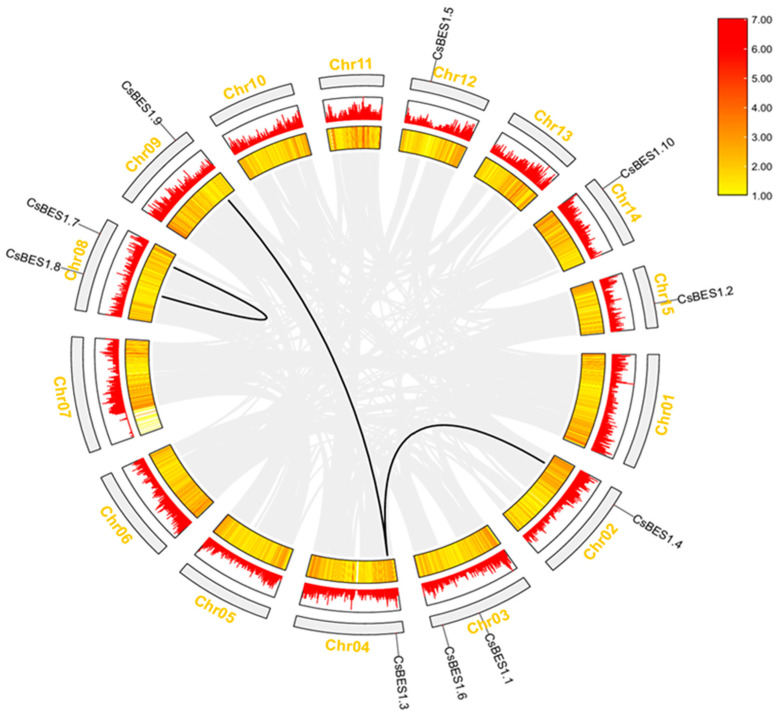
The distribution of *CsBES1* genes across 15 chromosomes, along with evidence of genomic amplification. A line plot and heatmap display the density of *CsBES1* genes along each chromosome. Black lines indicate gene pairs originating from *CsBES1*.

*TGY* tea has a total of 12 chromosomes, with 10 *CsBES1* genes distributed across 8 of them. Chromosomes 8 and 3 contain the highest number of *CsBES1* members, as shown in Figure 4. Specifically, *CsBES1.7* and *CsBES1.8* are located on chromosome 8, while *CsBES1.1* and *CsBES1.6* are found on chromosome 3. The remaining *CsBES1* genes are distributed singly across chromosomes 9 (*CsBES1.9*), 12 (*CsBES1.5*), 14 (*CsBES1.10*), 15 (*CsBES1.2*), 2 (*CsBES1.4*), and 4 (*CsBES1.3*). This uneven distribution pattern likely reflects genetic variations that occurred during evolution. The collinear model provides valuable insights into the evolutionary history of the genome and facilitates subsequent correlation analyses [68]. Notably, three pairs of collinear genes were identified: one tandem duplication (*CsBES1.7* and *CsBES1.8*) and two segmental duplications (*CsBES1.9* and *CsBES1.3*, *CsBES1.4* and *CsBES1.3*).

**Figure 5:**
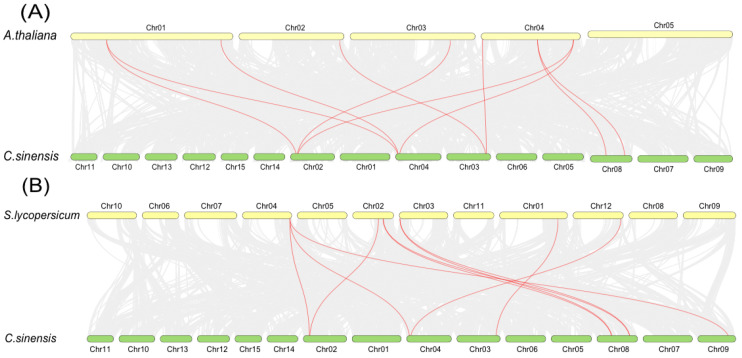
Comparative synteny analysis of *BES1* genes across *TGY*, *A. thaliana* (**A**), and *S. lycopersicum* (**B**). Grey lines in the background highlight syntenic *BES1* gene pairs, while red lines indicate collinearity between *CsBES1* and genes from other species. Chromosome numbers are displayed at the top or bottom of their respective diagrams.

Gene duplications, whether tandem (within a 200 kb region) or segmental (between different chromosomes), are crucial drivers of evolutionary innovation and functional diversification [41,69,70]. To gain deeper insights into the evolution of the *BES1* gene family, interspecific and intraspecific collinearity analyses were conducted (Figure 5). For interspecific analysis, *A. thaliana*, *S. lycopersicum*, and *TGY* were selected as study subjects to examine the consistency of their gene sequences. The results revealed 10 collinear gene pairs between *TGY* and each of the other two species, with chromosome 8 in *TGY* exhibiting the highest number of collinear pairs. Specifically, *TGY* shares 4 collinear pairs with *S. lycopersicum* and 2 pairs with *A. thaliana*. Across the three species, a total of 20 collinear genes were identified, suggesting conserved evolutionary roles.

### 2.5. Cis-Regulatory Element Prediction of Promoters in 10 CsBES1 Genes

**Figure 6:**
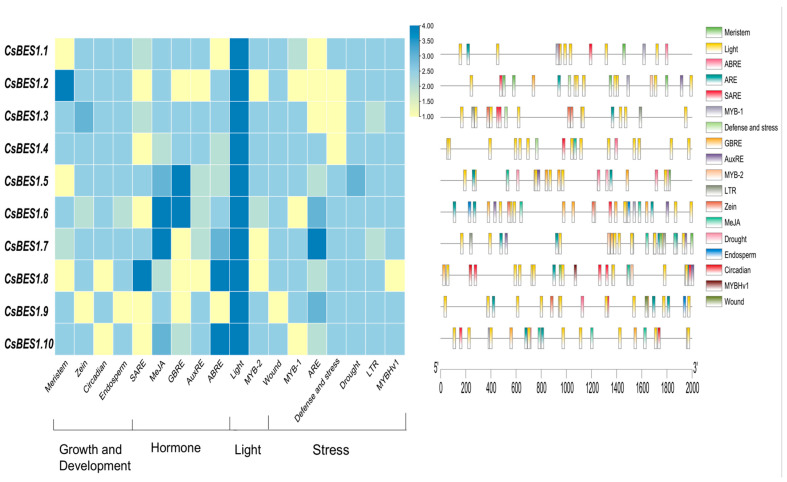
Schematic representation and visualization of the localization of cis-acting elements in each *CsBES1* gene.

Transcription factors (TFs) mainly exert their regulatory function by binding to specific cis-acting elements found in the promoter regions of target genes. This interaction regulates gene expression and influences the expression patterns of downstream genes [71]. In this study, the 2.0 kb promoter sequences of *CsBES1* genes were analyzed using the PlantCARE online software, which identified several cis-acting elements [72]. Based on their functional characteristics, these elements were categorized into four groups (Figure 6): hormonal response, stress signaling, light signaling, and growth and development.

The *CsBES1* genes showed significant correlations with hormone response elements, particularly ABA response elements (ABRE), salicylic acid response elements (SARE), methyl jasmonate (MeJA) response elements, gibberellin response elements (GBRE), and auxin response elements (AuxRE). This indicates a possible link between the *CsBES1* gene family and the signaling pathways of ABA and growth hormones, which is consistent with prior studies [14,73]. Additionally, these genes respond to various external stressors, including drought (e.g., MYB-1, MYBHv1, and other drought-related factors), low temperature (LTR), wounding and defense (WUN), and hypoxia (ARE). Nearly all *CsBES1* genes contain MYB-like transcription factor binding sites, which are strongly associated with drought response. MYB-like TFs are critical for regulating abiotic stress responses, especially drought stress [74]. These findings underscore the significant role of the *CsBES1* gene family in enhancing plant resistance to drought stress.

Notably, two types of light-responsive cis-elements were detected, making them the most common cis-elements in the *CsBES1* promoter regions. *CsBES1.4* was found to contain 13 light-related cis-elements, the highest among all family members. *CsBES1.9* and *CsBES1.10* were found to contain 12 light-related cis-elements. In addition, *CsBES1.2*, *CsBES1.7,* and *CsBES1.8* have only one photo-responsive homeostatic element. Several *CsBES1* genes contain cis-elements that are associated with various aspects of plant growth and development. These include elements linked to mesophyll expression (MREs), components involved in zein metabolism, regulatory elements for circadian rhythm (Circadian), and those specific to endosperm expression. Genes such as *CsBES1.3*, *CsBES1.4*, *CsBES1.5*, *CsBES1.6*, and *CsBES1.7*, which contain diverse regulatory elements, are likely to play critical roles in responding to abiotic stresses. These family members merit further investigation.

### 2.6. Gene Ontology (GO) and KEGG Analysis in CsBES1

**Figure 7:**
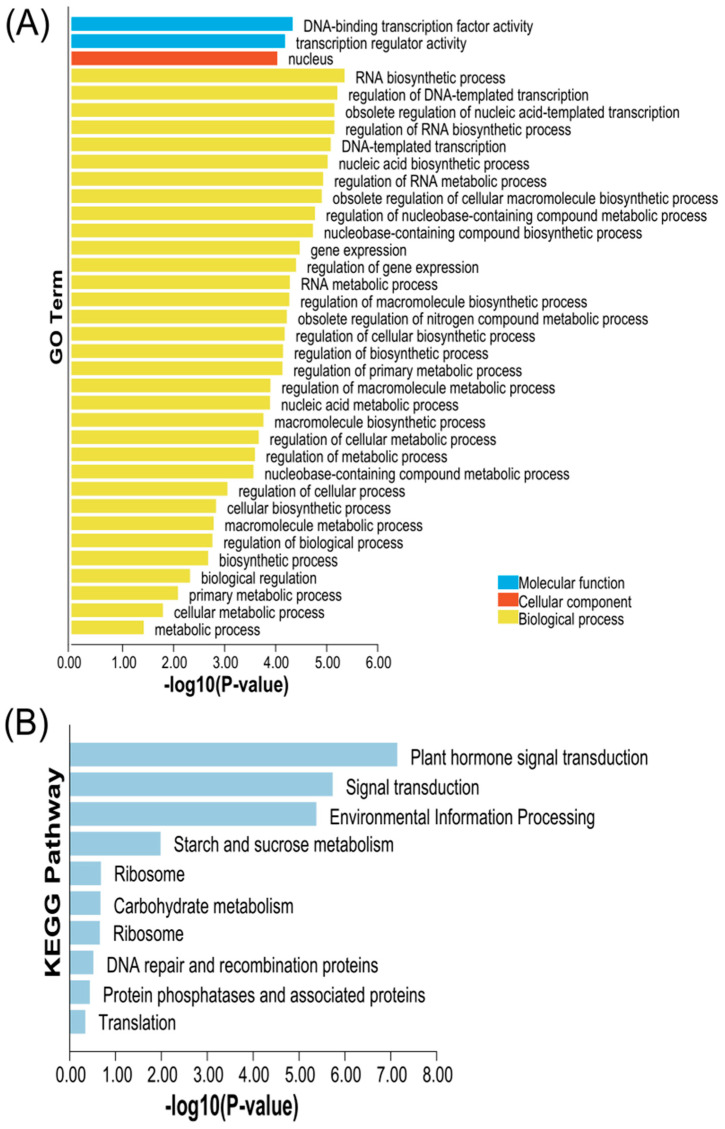
GO enrichment (**A**) and KEGG pathway and (**B**) analysis of *BES1* genes.

In order to gain deeper insights into the functions of *CsBES1* genes, a Gene Ontology (GO) enrichment analysis was performed (Figure 7A). This analysis covered three main categories: molecular function, cellular components, and biological processes. This analysis provides valuable insights and a foundation for future exploration of the functions and mechanisms of *CsBES1* genes. Notably, *CsBES1* genes may interact with DNA-binding TF activity and transcription regulator activity. Furthermore, these genes are predominantly localized in the nucleus, aligning with subcellular localization predictions. Additionally, *CsBES1* genes collectively contribute to the biosynthesis of various secondary metabolites and participate in plant hormone signaling pathways (Figure 7B).

### 2.7. Protein–Protein Interaction Network of CsBES1 Proteins

**Figure 8:**
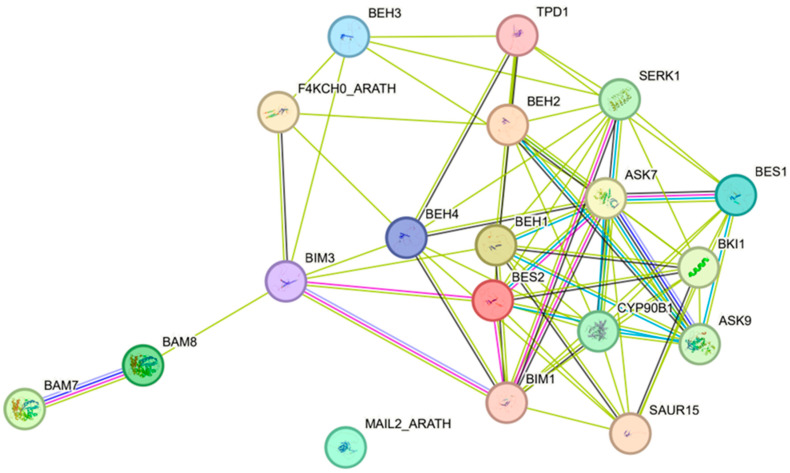
Protein–protein interaction network generated using STRING. The size of each protein’s circle corresponds to its number of interactions, with larger circles and deeper colors indicating higher correlation.

Alignment of *CsBES1* genes with *AtBES1* genes identified ten homologous genes, offering insights into potential functional interactions between *CsBES1*-encoded proteins and other key regulatory proteins. This understanding establishes a foundation for future functional validation and mechanistic studies. The interaction network of *CsBES1* proteins was constructed using STRING and refined in Cytoscape (Figure 8). *BES1*, a plant-specific TF, is a critical regulator of plant embryonic development. Homologous proteins BEH1, BEH2, BEH3, and BEH4 function alongside *BES1* to regulate plant growth and development by modulating downstream gene expression [75]. In the interaction network, *BES1* serves as a central node, linking with other proteins to form a complex signal transduction network. It acts as a downstream TF in the TPD1-SERK1 signaling pathway, which regulates tapetum development and plays a vital role in plant reproduction [17].

*BES1* is also predicted to interact with additional signaling components, including CYP90B1, SAUR15, ASK7, and BKI1; this process plays a crucial role in controlling and influencing the growth and development of plants. Furthermore, the probable interactions of *BES1* with SAUR15, ASK7, BKI1, and SCRM suggest a role for CsBES1 in helping plants cope with abiotic stress [76,77]. CYP90B1, indirectly associated with *BES1*, is an enzyme from the cytochrome P450 family involved in diverse metabolic processes, highlighting its potential role in growth and development [78]. A deep understanding of the functional interactions between *CsBES1* proteins and essential regulatory proteins establishes a solid basis for subsequent investigations into their roles and mechanisms.

### 2.8. Expression Patterns of CsBES1 Genes in Response to Dark and Light Treatments

**Figure 9:**
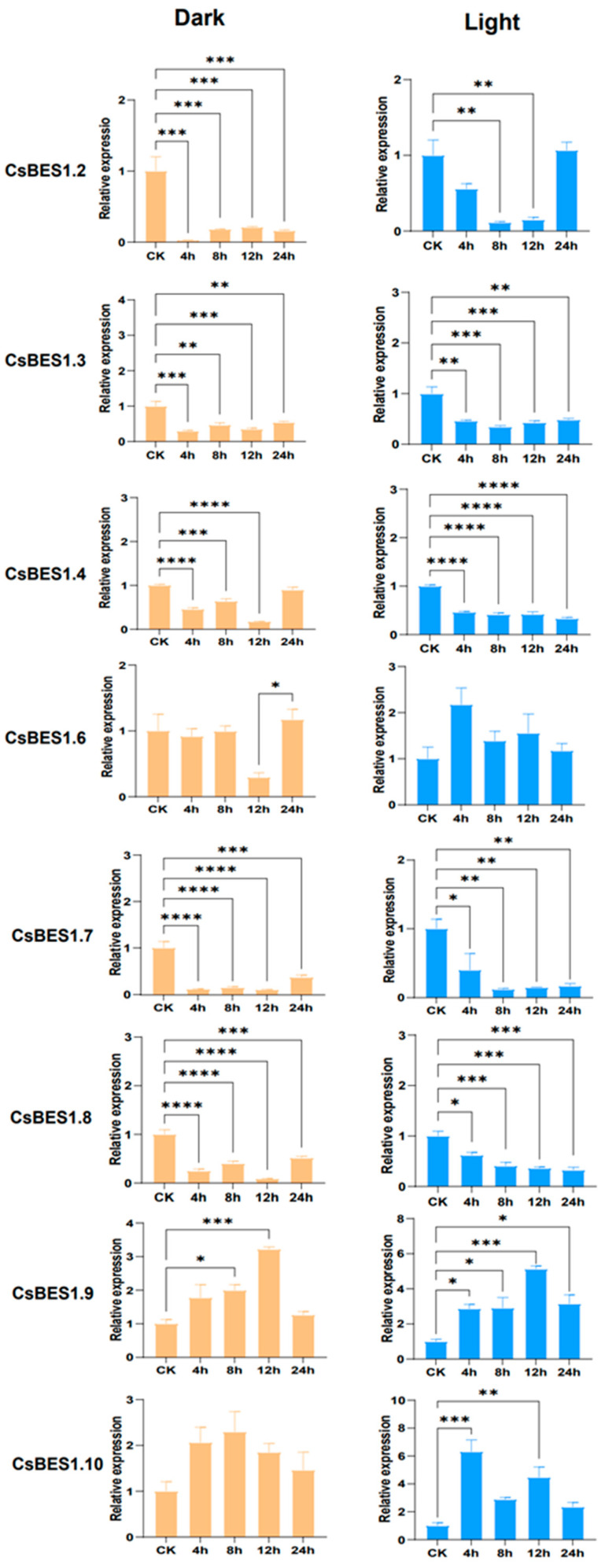
Expression profiles of *CsBES1* genes under various stress conditions. Error bars represent the standard deviation (SD). Statistical significance was assessed using one-way ANOVA, with the number of asterisks (*) indicating the level of significance (*p* < 0.05). Significant differences relative to the control group are indicated as follows: * *p* < 0.05, ** *p* < 0.01, *** *p* < 0.0005, and **** *p* < 0.0001.

Earlier research has thoroughly investigated the fundamental roles of the *BES1* gene family in response to abiotic stress. In the present study, we examined the expression profiles of eight *CsBES1* genes, categorized into four subgroups (I–IIIB), under two different abiotic stress conditions using quantitative real-time PCR (qRT-PCR). Stress treatments included light exposure (continuous 1200 µmol∙m^−2^∙s^−1^) and darkness. And the TmThe expression patterns of *CsBES1* genes exhibited similar responses to abiotic stresses, with some genes showing significant upregulation or downregulation under specific conditions (Figure 9).

Under dark treatment, *CsBES1.2*, *CsBES1.3*, *CsBES1.4*, *CsBES1.7*, and *CsBES1.8* displayed significant downregulation after four hours of treatment. Notably, *CsBES1.4*, *CsBES1.7*, and *CsBES1.8* showed pronounced downregulation (*p* < 0.0001), reaching their lowest expression levels after 12 h. Subsequently, their expression levels began to increase as the duration of dark treatment was prolonged. In contrast, *CsBES1.9* and *CsBES1.10* exhibited upregulation after 12 h, with *CsBES1.9* demonstrating a three-fold increase compared to the control group, suggesting that *CsBES1.9* may be particularly sensitive to light reduction. However, the underlying regulatory mechanism remains unclear and requires further investigation.

Under light exposure, *CsBES1.9* and *CsBES1.10* showed significant upregulation, with *CsBES1.10* reaching approximately six-fold the control expression level after four hours. After eight hours, the expression of all genes was inhibited except for *CsBES1.9*, which continued to upregulate and peaked at 12 h. Conversely, the expression of *CsBES1.2* and *CsBES1.7* became nearly undetectable. Notably, *CsBES1.9* exhibited significant upregulation after 12 h of both darkness and light exposure (*p* < 0.0005), while *CsBES1.4* showed significant downregulation under both conditions after 12 h (*p* < 0.0001). These findings suggest that *CsBES1.9* and *CsBES1.4* may have critical roles in responding to abiotic stress. Furthermore, it is speculated that responses to light and dark stress in ferroptosis are primarily regulated by a few specific *CsBES1* genes.

## 3. Discussion

Tea, as a perennial woody plant, is widely distributed and exhibits significant resource diversity. Over its evolutionary history, the tea plant has developed specialized mechanisms to adapt to environmental changes. One critical area requiring further investigation is the molecular mechanism by which *CsBES1* regulates the tea plant’s resistance to light stress. *BES1* family members are widely distributed across plants and are essential for regulating BR signaling pathways. These proteins are involved in various processes, including responses to abiotic stress, cell proliferation and differentiation, seed germination, trichome cell degradation, and pollen development. This study identified 10 *BES1* genes using bioinformatics methods, fewer than the 14 identified in *Populus tomentosa* [41], but more than the 6 identified in rice [79], and comparable to the counts in grape (8) [80] and *Machilus nanmu* (9) [81].

Analysis of the physicochemical properties of *CsBES1* revealed that it is an unstable, hydrophilic protein capable of functioning in mildly acidic and alkaline environments. Consistent with these findings, the subcellular localization of *AtBES1* has been observed in both the nucleus and cytoplasm [26,34]. Similarly, in our investigation, we found that the 10 genes are distributed across the nucleus and cytoplasm (Table 1), suggesting that *TGY BES1* proteins participate in phosphorylation-regulated processes.

To explore the evolutionary relationships among *Arabidopsis thaliana*, *Solanum lycopersicum*, and *TGY*, we constructed a phylogenetic tree for the *CsBES1* protein family. This analysis revealed that the 33 *BES1* proteins are classified into four subgroups, with the majority of *CsBES1* genes residing in subgroup IIIB (Figure 3). Previous studies have shown that changes in gene family size can lead to beneficial, harmful, or neutral outcomes and that variations in gene family numbers significantly influence species specificity [79]. Our findings align with this, demonstrating that the phylogenetic relationships of *BES1* genes differ among species.

Additionally, *SlBES1.8*, a key regulator of fruit setting and development in *S. lycopersicum* is induced by auxin and gibberellin [19]. Since *CsBES1.1* is a direct homolog of *SlBES1.8* in subgroup IIIB, it is plausible that *CsBES1.1* also contributes to *TGY* development. Interestingly, motif analysis (Figure 2) reveals that *CsBES1.1* is approximately 25 kb in length, making it the longest gene in this family. Given the established relationship between gene structure and function [80], further investigation into *CsBES1.1* is of significant scientific interest. Furthermore, *CsBES1.2* and *CsBES1.5* cluster with *SlBES1.8*, suggesting their potential involvement in related regulatory processes. It is also noteworthy that *BES1* genes in Class I and II groups are exclusively found in *Arabidopsis thaliana*, indicating that *BES1* genes in *TGY* may have undergone expansion following the divergence of monocots.

Martin’s analysis suggests that *BES1* first appeared in bryophytes and evolved further after phytoplankton [67]. Previous studies have shown that *AtBZR1* plays a redundant role in plant growth and development by facilitating BR-induced growth while providing feedback inhibition on BR biosynthesis [81,82]. In contrast, *AtBES1* is involved in glucosinolate biosynthesis via BR signaling [26]. Our study identified that *CsBES1.10* clusters with *AtBES1.13-14*, while *CsBES1.6* forms an orthologous gene pair with *AtBES1.9*, both potentially involved in related regulatory processes.

To further investigate the evolution of the *BES1* gene family, we performed both inter- and intra-species synteny analyses, which revealed 20 pairs of syntenic genes across the three species, underscoring their conserved evolutionary significance.

Gene families mainly arise through six distinct mechanisms: whole-genome duplication, tandem duplication, segmental duplication, insertion of retrotransposons, exon duplication, and reshuffling [69]. The expansion of the *CsBES1* gene family primarily occurs through fragment replication events, which represent its primary evolutionary mechanism. Similarly, the *BES1* gene family in wheat expands through segmental duplication [83]. In species such as *A. thaliana*, cabbage, rice, and apple, segmental duplications are predominantly observed among homologous *BES1* paralogous genes, aligning with our findings in *TGY* [84,85]. These results underscore the importance of segmental duplication in the amplification of *CsBES1* genes and highlight the gene family’s critical role in understanding *TGY*’s biological evolution.

Introns are crucial in plant evolution [86]. Exon-intron structure analysis further revealed that *CsBES1.2* and *CsBES1.5* lack non-coding regions, which are crucial for regulating mRNA stability and gene expression. A phylogenetic tree constructed for the *BES1* gene family indicated a clear evolutionary relationship between *CsBES1* genes in ‘*Tieguanyin*’ tea, *Arabidopsis thaliana*, and *Solanum lycopersicum*. Specifically, *CsBES1.2* and *CsBES1.5* clustered with *SlBES1.8* from tomato, which is known to play a key role in fruit set and development in tomato and is induced by auxin and gibberellin. This suggests that *CsBES1.1*, as a direct homolog of *SlBES1.8*, may play a similar role in the development of ‘*Tieguanyin*’ tea. Furthermore, *CsBES1.10* clustered with *AtBES1.13* and *AtBES1.14* from *Arabidopsis*, while *CsBES1.6* is homologous to *AtBES1*.9, possibly participating in similar regulatory pathways.

Exon–intron analysis identified 10 *CsBES1* genes, with *CsBES1.6*, *CsBES1.10*, *CsBES1.7*, and *CsBES1.8* showing conserved motifs and similar structures. Phylogenetic analysis supports these findings. *CsBES1.2* and *CsBES1.5* lack non-coding regions, unlike the grape *BES1* family [87], which includes regulatory non-coding regions affecting mRNA stability and gene expression. Most *CsBES1* proteins contain motif 1, the most conserved. These results highlight the importance of motifs 1 and 2 in the *CsBES1* gene family.

To further investigate the potential role of the *BES1* gene family in environmental responses, we analyzed the light-responsive cis-acting elements in the promoter regions of *CsBES1* genes. Cis-acting elements, which play a crucial role in the initiation of genes and the regulation of transcription, are found within the promoters [88]. The *BES1* gene family plays a critical role in various stress responses [13,42,89,90]. The results showed that *CsBES1.4* contains the highest number of light-related cis-acting elements (13), followed by *CsBES1.9* and *CsBES1.10*. QRT-PCR analysis revealed that under light conditions, the expression of *CsBES1.9* and *CsBES1.10* was significantly upregulated, with *CsBES1.10* reaching six times the control group’s expression level after 4 h. After 8 h, all genes except *CsBES1.9* showed suppressed expression, while *CsBES1.9* continued to increase and peaked at 12 h. In contrast, the expression of *CsBES1.2* and *CsBES1.7* was nearly undetectable. Notably, *CsBES1.9* exhibited significant upregulation after 12 h of both dark and light exposure (*p* < 0.0005), while *CsBES1.4* showed significant downregulation under both conditions (*p* < 0.0001). These results suggest that *CsBES1.9* and *CsBES1.4* may play key roles in responding to abiotic stress.

Using *A. thaliana* as a model, we predicted a protein–protein interaction network. As a key downstream TF in the TPD1-SERK1 signaling pathway, *BES1* plays an essential role in regulating tapetum layer development, thereby influencing plant reproduction. Building on previous findings, we identified eight *CsBES1* homologous genes and analyzed their expression patterns under different light conditions. Quantitative real-time polymerase chain reaction (qRT-PCR) analysis revealed that the upregulation of *CsBES1.9* and *CsBES1.10* under light exposure, along with the downregulation of *CsBES1.4*, *CsBES1.7*, and *CsBES1.8* under dark conditions, highlights the differential regulation of these genes in response to light and dark stress. It is speculated that the genes containing the most light-responsive cis-elements (*CsBES1.4*, *CsBES1.9*, and *CsBES1.10*) play critical roles in regulating light stress responses and ferroptosis processes in plants. These results suggest that these genes may play pivotal roles in responding to light variations and adapting to environmental stress.

**Figure 10:**
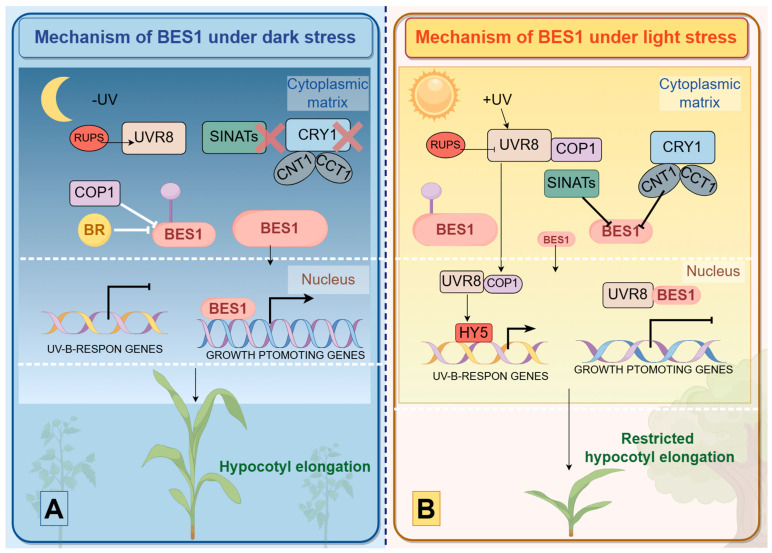
In darkness (**A**), CRY1 remains in its inactive form and is unable to suppress BR signaling because it does not interact with dephosphorylated *BES1*. SINATs undergo self-degradation, leading to a rise in the levels of dephosphorylated *BES1*, while phosphorylated *BES1* (*pBES1*) is degraded via the COP1 pathway. As a result, the ratio of dephosphorylated *BES1* to *pBES1* increases. The active dephosphorylated *BES1* then regulates gene expression to promote hypocotyl elongation. During this phase, UVR8 predominantly resides in the cytoplasm, whereas BIM1 and functional BES1 are localized in the nucleus, where they facilitate the transcription of BR-induced genes to enhance hypocotyl growth. The meaning of ‘X’ is not to express it in this situation. In light conditions (**B**), CRY1 becomes activated and binds to dephosphorylated *BES1*, inhibiting its ability to bind DNA. The accumulation of SINATs promotes the degradation of *BES1*, resulting in a decrease in dephosphorylated *BES1*/*BZR1* levels and reducing the ratio of dephosphorylated *BES1*/*BZR1* to phosphorylated *BES1*/*BZR1* (*pBES1*/*pBZR1*). This reduction in the ratio leads to the suppression of hypocotyl elongation. Under UV-B light, UVR8 accumulates in the nucleus, where it interacts with nuclear BIM1 and dephosphorylated *BES1*, blocking their DNA-binding activity and thereby repressing the transcription of BR-responsive genes and hypocotyl elongation. The varying font sizes for *BES1* indicate different levels of dephosphorylated and phosphorylated *BES1*.

*BES1*/*BZR1* plays a central role in regulating light signaling during plant morphogenesis, with its phosphorylation status and stability modulated by light signals [63]. Previous studies have shown that under dark stress, the protein levels of SINATs decrease, leading to the activation of COP1 [91]. Simultaneously, inactive CRY1 fails to interact with dephosphorylated *BES1*, resulting in elevated levels of dephosphorylated *BES1*. This dephosphorylated form can form homodimers (*BES1-BES1* and *BZR1*-*BZR1*) and heterodimers (*BES1*-*PIF4* and *BZR1-PIF4*) to regulate downstream gene expression, promoting hypocotyl elongation (Figure 10A) [92]. It is important to note that Constitutive Photomorphogenic 1 (COP1), an E3 ubiquitin ligase, is responsible for the dark-induced degradation of the inactive, phosphorylated version of *BZR1* [64].

In the presence of light, SINAT levels rise, facilitating the degradation of dephosphorylated *BES1*/*BZR1*. At the same time, COP1 activity is inhibited, leading to a decrease in the ratio of *BES1*/*BZR1* to p*BES1*/p*BZR1* [91]. Additionally, activated CRY1 interacts with dephosphorylated *BES1* through its N-terminal (CNT1), inhibiting its DNA-binding activity and suppressing hypocotyl growth (Figure 10B) [93]. UV-B light, an integral component of sunlight, significantly affects plant development via the UVR8 photoreceptor [94,95,96,97]. Under UV-B exposure, UVR8 interacts with COP1 and accumulates in the nucleus, where it binds to dephosphorylated *BES1*. This interaction inhibits *BES1*’s DNA-binding activity, reducing the transcription of growth-promoting genes and thereby suppressing hypocotyl elongation [97,98,99].

When UV-B radiation is not present, UVR8 remains mainly in the cytoplasm, whereas BIM1 and active *BES1* are localized in the nucleus, where they facilitate the transcription of genes induced by brassinosteroids (BR). This activity enhances cell elongation under low-temperature conditions. Our analysis of cis-acting elements and protein–protein interactions (Figure 5 and Figure 7) suggests a potential link between key response proteins and *CsBES1*, indicating that *CsBES1* may play a crucial role in environmental stress resistance by interacting with or regulating these proteins (Figure 10). While these findings align with our research on *CsBES1* and previous studies on plant stress responses, further experiments are necessary to confirm its precise contributions to plant resilience.

## 4. Materials and Methods

### 4.1. Identification and Analysis of BES1 Gene Families in TGY

*TGY* was acquired from the China National GeneBank genome sequence database sequence file (https://db.cngb.org/search/project/CNP0002030/ (accessed on 1 October 2024)) [100]. Meanwhile, amino acid sequence information of *A. thaliana BES1* was obtained from the TAIR database (https://www.arabidopsis.org (accessed on 1 October 2024)). The protein sequence of *TGY* was compared with *AtBES1*s using the BLASTp 2.12.0 program of the National Center for Biotechnology Information (NCBI). Combining the results obtained using these two methods, *CsBES1*s was accurately located. Then, we used the NCBI CDD search tool (https://www.ncbi.nlm.nih.gov/Structure/bwrpsb/bwrpsb.cgi (accessed on 2 October 2024)) and SMART network database (http://smart.Embl-heidelberg.de/ (accessed on 3 October 2024)) to carry out in-depth analysis and verification of *CsBES1*s.

Finally, Tie Guanyin’s *BES1* gene family was successfully identified as having 10 gene family members, and it was renamed *CsBES11-10*. ExPASy online (https://web.expasy.org/protparam (accessed on 15 July 2024)) [101] was utilized to predict and calculate the molecular weight, number of amino acids, theoretical PI (isoelectric point), number of positive (negative) residues, instability coefficient, lipolysis coefficient, and total average hydrophilicity of each *BES1* protein in *TGY*.

### 4.2. Physical and Chemical Properties of TGY

HMMER 3.3 software was used to conduct comprehensive screening of the *TGY* genome and accurately locate *the BES1* homologous sequence. The preliminary screening of the proceeds of the gene sequences was uploaded to the SMART database (https://smart.embl-Heidelberg.DE/ (accessed on 4 October 2024)) and the National Center for Biotechnology Information (NCBI) website (https://www.ncbi.nlm.nih.gov/ (accessed on 8 October 2024)). In this way, its domain can be accurately confirmed. In this process, the redundant sequences were effectively eliminated through meticulous manual comparison and screening, so as to successfully obtain *CsBES1* gene members. Subsequently, the physicochemical properties of *CsBES1* gene members were further investigated using the Protparam database (https://www.expasy.org/ (accessed on 9 October 2024)).

The ProtParam tool available on the ExPasy website was used to perform an in-depth analysis of essential protein characteristics, including the number of amino acids, molecular weight, and isoelectric point for each member. This analysis provided valuable data and a theoretical foundation for a deeper understanding of the properties and functions of the *CsBES1* gene family. Furthermore, the CDD function of the NCBI website (https://www.ncbi.nlm.nih.gov/cdd (accessed on 10 October 2024)) and SMART online (https://smart.embl.de/ (accessed on 11 October 2024)) [31] were employed to analyze and integrate the obtained protein sequences.

### 4.3. Phylogenetic Analysis of CsBES1s and BES1s from Other Species

In this study, the *BES1* protein sequence of *TGY* was successfully obtained by accessing the Ensemble Plant database. For *Arabidopsis* and *S. lycopersicum*, the genome and annotated data were downloaded from the specific ftp (https://www.etc.site/ftp/genomes/Nicotiana_tabacum/edwards_et_al_2017/ (accessed on 13 October 2024)). In order to deeply explore the evolutionary relationship between different species, the phylogenetic tree was constructed by adopting the neighbor-joining method (NJ) and setting 1000 repeats (the bootstrap value was 1000). Firstly, the online tool ITOL (https://itol.embl.de/ (accessed on 14 October 2024)) was used to construct preliminary phylogenetic trees for *Arabidopsis* and *S. lycopersicum* and *TGY* species, and MEGA 7.0.26 software was applied to construct the phylogenetic trees.

### 4.4. Collinearity Analysis and Replication Event Analysis of BES1

We used MCScanX (https://github.com/wyp1125/MCScanX/ (accessed on 15 October 2024)) software to study *Arabidopsis* and rice and maize *CsBES1* and *BES1* gene synthesis relationship in depth [68]. With the help of this software, the segmentation and tandem replication events occurring in the *CsBES1* gene were identified, providing key genetic structural variation information. At the same time, TBtools-v2.10 [102], a software tool, was utilized to conduct a comprehensive analysis of the visual distribution and collinearity of *CsBES1* genes.

### 4.5. GO and KEGG Analysis

In this study, the egg NOG-MAPPER platform was used to implement GO and KEGG analysis processes for the *BES1* protein (https://www.kegg.jp/ (accessed on 20 October 2024)) [103]. In the framework of this analysis, the number of *CsBES1* proteins was subdivided into three categories [104]. Then, Tbtools v2.10 software was used to visualize and analyze the annotation results.

### 4.6. Protein–Protein Interaction Network of CsBES1s

Complete and in-depth analysis is performed by uploading the *BES1* protein sequence to the STRING database (https://cn.string-db.org/ (accessed on 25 October 2024)). Based on the observations of protein interactions in Arabidopsis, the association patterns among key proteins were prospectively predicted. Finally, Cytoscape (V3.10.2) software is used to visualize and optimize the acquired network [105].

### 4.7. Abiotic Stress Therapy

In this experiment, *TGY* seedlings were selected from *TGY* specimens. The soil for planting seedlings was prepared using a mixture of peat, humus, sandy soil, and perlite in a ratio of 5:2:2:1, resulting in soil organic content ranging from 2.57% to 6.07%. The average annual temperature was 16–20 °C, the annual precipitation was 900–2100 mm, and the annual relative humidity was about 77%. There were three experimental groups, drought, heat stress, and light treatment, and each experimental group included several groups. The drought and heat stress groups were divided into 4 groups, and the light stress groups were divided into 3 groups. Each group was treated with customized experimental conditions. In the sampling time setting, the sampling time of the drought and heat treatment groups was 0, 4, 8, 12, and 24 h.

The Nt group was sampled at 0, 24, 48, and 72 h, and seedlings collected at 0 h were used as the control group. During stress treatment, the parameters of the artificial climate chamber were set as follows: photocycle was 12 h/day, LED lighting was used, photosynthetic activity E radiation was set at 1200 µmol·mol^−1^·s^−1^, and the temperature was constant at 25 °C. Drought stress simulation was carried out with a specific scheme. The seedlings were incubated at 40 °C during temperature treatment. For light treatment, the corresponding treatment group was exposed to a light intensity of 1200 µmol·mol^−1^·s^−1^ for 24, 48, and 72 h, while the control group was exposed to 12 h of dark treatment and sampled at the half photocycle time. After processing, leaf specimens were quickly collected and stored in liquid nitrogen at −80 °C for subsequent RNA extraction.

Real-time PCR analysis was performed with 1 μL cDNA template, 10 μL SYBR Premix-Ex TaqTM II, 2 μL specific primers, and 7 μL ddH_2_O. The reaction conditions were as follows: 95 °C for 30 s; 95 °C for 5 s; 60 °C for 30 s; 95 °C for 5 s; 60 °C for 60 s; and 50 °C for 30 s, for a total of 40 cycles. The internal reference gene was *GAPDH* (GenBank No. KX682032) [106]. The expression level of the target gene was calculated using the 2-AACt method, and the quantitative data were analyzed with the *t*-test in SPSS26. Statistical significance was determined when *p* < 0.05. GraphPad Prism8.0 was applied for data visualization. Table 2 lists the primer sequences used, with *GAPDH* serving as the reference gene.

### 4.8. RNA Extraction and Statistical Analysis

For the RNA extraction and cDNA synthesis experiment, we used Omega Bio-Tek’s RNA extraction (Norcross, GA, USA) kit to extract total RNA from the sample. RNA extracted from Transgen was used as a template to synthesize cDNA for subsequent experiments. For the qRT-PCR experiment, the materials required are 1 μL cDNA, 2 μL specific primers (concentration 0.5 μM), 10 μL SYBR Premix Ex TaqTM II, and 7 μL ddH_2_O. The reaction process is denaturation annealing and extension. Finally, the relative expression of the *CsBES1* gene was calculated using 2^−∆∆CT^ method, and one-way variance analysis and Duncan multiple comparison tests were performed using GraphPad Prism 9.0 software (software web site: https://www.graphpad.com/ (accessed on 1 November 2024)) [107]. All quantitative PCR experiments were repeated three times.

## 5. Conclusions

In this study, a total of 10 *CsBES1* genes were identified in *TGY*, and their properties, structural features, functional roles, relationships, and expression profiles were thoroughly analyzed. Among them, *CsBES1.3*, *CsBES1.4*, *CsBES1.5*, *CsBES1.6*, and *CsBES1.7*, which contain promoter regions, are more likely to play critical roles in responding to various abiotic stresses. The *BES1* proteins may interact with other signal transduction components, such as CYP90B1, SAUR15, ASK7, and BKI1, to regulate plant growth and development. Further analysis revealed that *CsBES1.9* and *CsBES1.4* exhibit the most significant expression changes under different stress conditions.

Specifically, the expression of *CsBES1.9* was most strongly upregulated under light stress, suggesting its potential role in light stress resistance. Nevertheless, the exact mechanisms by which *CsBES1* genes control the growth and development of *TGY* remain unclear and warrant further exploration. This study offers a comprehensive analysis of the expression patterns of *CsBES1* genes across various tissues and their responses to abiotic stress, shedding light on their potential roles in plant stress tolerance.

## Figures and Tables

**Table 1:** Physical and chemical characteristics of the ‘*Tieguanyin*’ *BES1*s including Gene Name, Gene ID, Size/aa (Size in Amino Acids), Molecular Weight/kDa (Molecular Weight in Kilodaltons), Theoretical PI (Theoretical Isoelectric Point), Grand Average of Hydropathicity, Instability Index, and Subcellular Localization.

Gene Name	Gene ID	Size/aa (Size in Amino Acids)	Molecular Weight/kDa (Molecular Weight in Kilodaltons)	Theoretical PI (Theoretical Isoelectric Point)	Grand Average of Hydropathicity	Instability Index	Subcellular Localization
*CsBES1.1*	*CsTGY03G0001903*	495	55,357.08	10.02	−0.765	72.8	Nucleus
*CsBES1.2*	*CsTGY15G0001195*	395	44,896.92	9.05	−0.391	43.17	Nucleus
*CsBES1.3*	*CsTGY04G0000351*	377	40,575.01	8.92	−0.654	81.82	Nucleus
*CsBES1.4*	*CsTGY02G0000736*	322	34,442.06	8.6	−0.608	70.99	Nucleus
*CsBES1.5*	*CsTGY12G0000463*	315	35,172.59	8.13	−0.604	37.02	Chloroplast
*CsBES1.6*	*CsTGY03G0002935*	699	78,007.57	5.37	−0.454	37.34	Nucleus
*CsBES1.7*	*CsTGY08G0002091*	323	34,725.64	7.61	−0.603	58.53	nucleus
*CsBES1.8*	*CsTGY08G0000981*	326	34,687.46	8.81	−0.609	59.21	nucleus
*CsBES1.9*	*CsTGY09G0002593*	316	34,258.36	9.25	−0.708	76.49	nucleus
*CsBES1.10*	*CsTGY14G0000640*	683	77,043.46	5.96	−0.511	40.49	nucleus

**Table 2:** Primer sequences used for qRT-PCR analysis of each *CsBES1* genes.

Gene Name	Forward Primer (5′-3′)	Reverse Primer (5′-3′)
*CsBES1.2*	GGGTTAAGCAGATGGGTGGT	GTTCCGGTGAGGTCGGAATG
*CsBES1.3*	GGAGGAGGGAGGAGGAAGCC	GTGCCATCGGGTTCGACTGT
*CsBES1.4*	CAAACACTGCGACAACAACG	CGATAAGTGGTGCCATCGTC
*CsBES1.6*	TGTGGAGGCAACGTTGGTGA	ATTCAGGGTTGCGCCTTCCC
*CsBES1.7*	TGGACTCCTGGGCAAAGTGG	TCCCATGCCTTCACCAACCC
*CsBES1.8*	GGGTCAGCTTCAGCAAGTCC	TGGCAGCGTAACGTGATGAG
*CsBES1.9*	CGCCAACTATGGTCCCAACC	GCTTCACTGGGACGCTTTCA
*CsBES1.10*	GTCAGGGCTGGTTCCGTGTC	ATCCTCGATCCCAGGCCGAA
*GAPDH*	TTGGCATCGTTGAGGGTCT	CAGTGGGAACACGGAAAGC

## Data Availability

Data are contained within the article.

## References

[1] Li L., Deng X.W. (2005). It runs in the family: Regulation of brassinosteroid signaling by the *BZR1-BES1* class of transcription factors. Trends Plant Sci..

[2] Wang Z.Y., Bai M.Y., Oh E., Zhu J.Y. (2012). Brassinosteroid signaling network and regulation of photomorphogenesis. Annu. Rev. Genet..

[3] Anwar A., Liu Y., Dong R., Bai L., Yu X., Li Y. (2018). The physiological and molecular mechanism of brassinosteroid in response to stress: A review. Biol. Res..

[4] Feng Z., Shi H., Lv M., Ma Y., Li J. (2021). Protein farnesylation negatively regulates brassinosteroid signaling via reducing *BES1* stability in Arabidopsis thaliana. J. Integr. Plant Biol..

[5] Li Q., Xu F., Chen Z., Teng Z., Sun K., Li X., Yu J., Zhang G., Liang Y., Huang X. (2021). Synergistic interplay of ABA and BR signal in regulating plant growth and adaptation. Nat. Plants.

[6] González-García M.P., Vilarrasa-Blasi J., Zhiponova M., Divol F., Mora-García S., Russinova E., Caño-Delgado A.I. (2011). Brassinosteroids control meristem size by promoting cell cycle progression in Arabidopsis roots. Development.

[7] Planas-Riverola A., Gupta A., Betegón-Putze I., Bosch N., Ibañes M., Caño-Delgado A.I. (2019). Brassinosteroid signaling in plant development and adaptation to stress. Development.

[8] Mitchell J.W., Mandava N., Worley J.F., Plimmer J.R., Smith M.V. (1970). Brassins--a new family of plant hormones from rape pollen. Nature.

[9] Peres A., Soares J.S., Tavares R.G., Righetto G., Zullo M.A.T., Mandava N.B., Menossi M. (2019). Brassinosteroids, the Sixth Class of Phytohormones: A Molecular View from the Discovery to Hormonal Interactions in Plant Development and Stress Adaptation. Int. J. Mol. Sci..

[10] Nolan T.M., Vukašinović N., Hsu C.W., Zhang J., Vanhoutte I., Shahan R., Taylor I.W., Greenstreet L., Heitz M., Afanassiev A. (2023). Brassinosteroid gene regulatory networks at cellular resolution in the Arabidopsis root. Science.

[11] Kim E.J., Russinova E. (2020). Brassinosteroid signalling. Curr. Biol..

[12] Jiang H., Tang B., Xie Z., Nolan T., Ye H., Song G.Y., Walley J., Yin Y. (2019). GSK3-like kinase BIN2 phosphorylates RD26 to potentiate drought signaling in Arabidopsis. Plant J..

[13] Ye H., Liu S., Tang B., Chen J., Xie Z., Nolan T.M., Jiang H., Guo H., Lin H.-Y., Li L. (2017). RD26 mediates crosstalk between drought and brassinosteroid signalling pathways. Nat. Commun..

[14] Bulgakov V.P., Avramenko T.V. (2020). Linking Brassinosteroid and ABA Signaling in the Context of Stress Acclimation. Int. J. Mol. Sci..

[15] Liu Z., Guo J.T., Li T., Xu Y. (2008). Structure-based prediction of transcription factor binding sites using a protein-DNA docking approach. Proteins Struct. Funct. Genet..

[16] Century K., Reuber T.L., Ratcliffe O.J. (2008). Regulating the regulators: The future prospects for transcription-factor-based agricultural biotechnology products. Plant Physiol..

[17] Chen W., Lv M., Wang Y., Wang P.A., Cui Y., Li M., Wang R., Gou X., Li J. (2019). *BES1* is activated by EMS1-TPD1-SERK1/2-mediated signaling to control tapetum development in Arabidopsis thaliana. Nat. Commun..

[18] Cui X.Y., Gao Y., Guo J., Yu T.F., Zheng W.J., Liu Y.W., Chen J., Xu Z.S., Ma Y.Z. (2019). BES/BZR Transcription Factor TaBZR2 Positively Regulates Drought Responses by Activation of TaGST1. Plant Physiol..

[19] Su D., Xiang W., Wen L., Lu W., Shi Y., Liu Y., Li Z. (2021). Genome-wide identification, characterization and expression analysis of *BES1* gene family in tomato. BMC Plant Biol..

[20] Kono A., Yin Y. (2020). Updates on BES1/BZR1 Regulatory Networks Coordinating Plant Growth and Stress Responses. Front. Plant Sci..

[21] Yin Y., Vafeados D., Tao Y., Yoshida S., Asami T., Chory J. (2005). A new class of transcription factors mediates brassinosteroid-regulated gene expression in Arabidopsis. Cell.

[22] Guo H., Ye H., Li L., Yin Y. (2009). A family of receptor-like kinases are regulated by *BES1* and involved in plant growth in Arabidopsis thaliana. Plant Signal Behav..

[23] Reinhold H., Soyk S., Simková K., Hostettler C., Marafino J., Mainiero S., Vaughan C.K., Monroe J.D., Zeeman S.C. (2011). β-amylase-like proteins function as transcription factors in Arabidopsis, controlling shoot growth and development. Plant Cell.

[24] Lachowiec J., Mason G.A., Schultz K., Queitsch C. (2018). Redundancy, Feedback, and Robustness in the Arabidopsis thaliana BZR/BEH Gene Family. Front. Genet..

[25] Otani Y., Tomonaga Y., Tokushige K., Kamimura M., Sasaki A., Nakamura Y., Nakamura T., Matsuo T., Okamoto S. (2020). Expression profiles of four *BES1/BZR1* homologous genes encoding bHLH transcription factors in Arabidopsis. J. Pestic. Sci..

[26] Yin Y., Wang Z.Y., Mora-Garcia S., Li J., Yoshida S., Asami T., Chory J. (2002). *BES1* accumulates in the nucleus in response to brassinosteroids to regulate gene expression and promote stem elongation. Cell.

[27] Mueller A.K., Labaied M., Kappe S.H., Matuschewski K. (2005). Genetically modified Plasmodium parasites as a protective experimental malaria vaccine. Nature.

[28] Li J., Wen J., Lease K.A., Doke J.T., Tax F.E., Walker J.C. (2002). BAK1, an Arabidopsis LRR receptor-like protein kinase, interacts with BRI1 and modulates brassinosteroid signaling. Cell.

[29] Kim T.W., Guan S., Burlingame A.L., Wang Z.Y. (2011). The CDG1 kinase mediates brassinosteroid signal transduction from BRI1 receptor kinase to *BSU1* phosphatase and GSK3-like kinase BIN2. Mol. Cell.

[30] He J.-X., Gendron J.M., Yang Y., Li J., Wang Z.-Y. (2002). The GSK3-like kinase BIN2 phosphorylates and destabilizes *BZR1*, a positive regulator of the brassinosteroid signaling pathway in Arabidopsis. Proc. Natl. Acad. Sci. USA.

[31] Li J., Nam K.H. (2002). Regulation of brassinosteroid signaling by a GSK3/SHAGGY-like kinase. Science.

[32] Toledo-Ortiz G., Huq E., Quail P.H. (2003). The Arabidopsis basic/helix-loop-helix transcription factor family. Plant Cell.

[33] Wang R., Wang R., Liu M., Yuan W., Zhao Z., Liu X., Peng Y., Yang X., Sun Y., Tang W. (2021). Nucleocytoplasmic trafficking and turnover mechanisms of BRASSINAZOLE RESISTANT1 in Arabidopsis thaliana. Proc. Natl. Acad. Sci. USA.

[34] Jiang J., Zhang C., Wang X. (2015). A recently evolved isoform of the transcription factor *BES1* promotes brassinosteroid signaling and development in Arabidopsis thaliana. Plant Cell.

[35] Saha G., Park J.I., Jung H.J., Ahmed N.U., Kayum M.A., Kang J.G., Nou I.S. (2015). Molecular characterization of BZR transcription factor family and abiotic stress induced expression profiling in Brassica rapa. Plant Physiol. Biochem..

[36] Setsungnern A., Muñoz P., Pérez-Llorca M., Müller M., Thiravetyan P., Munné-Bosch S. (2020). A defect in *BRI1-EMS-SUPPRESSOR 1* (*bes1*)-mediated brassinosteroid signaling increases photoinhibition and photo-oxidative stress during heat stress in Arabidopsis. Plant Sci..

[37] Yan M.Y., Xie D.L., Cao J.J., Xia X.J., Shi K., Zhou Y.H., Zhou J., Foyer C.H., Yu J.Q. (2020). Brassinosteroid-mediated reactive oxygen species are essential for tapetum degradation and pollen fertility in tomato. Plant J..

[38] Mecchia M.A., García-Hourquet M., Lozano-Elena F., Planas-Riverola A., Blasco-Escamez D., Marquès-Bueno M., Mora-García S., Caño-Delgado A.I. (2021). The *BES1*/*BZR1*-family transcription factor M*pBES1* regulates cell division and differentiation in Marchantia polymorpha. Curr. Biol..

[39] Korwin Krukowski P., Visentin I., Russo G., Minerdi D., Bendahmane A., Schubert A., Cardinale F. (2022). Transcriptome Analysis Points to *BES1* as a Transducer of Strigolactone Effects on Drought Memory in Arabidopsis thaliana. Plant Cell Physiol..

[40] Sun Z., Liu X., Zhu W., Lin H., Chen X., Li Y., Ye W., Yin Z. (2022). Molecular Traits and Functional Exploration of *BES1* Gene Family in Plants. Int. J. Mol. Sci..

[41] Yang J., Wu Y., Li L., Li C. (2022). Comprehensive analysis of the *BES1* gene family and its expression under abiotic stress and hormone treatment in Populus trichocarpa. Plant Physiol. Biochem..

[42] Chen J., Nolan T.M., Ye H., Zhang M., Tong H., Xin P., Chu J., Chu C., Li Z., Yin Y. (2017). Arabidopsis WRKY46, WRKY54, and WRKY70 Transcription Factors Are Involved in Brassinosteroid-Regulated Plant Growth and Drought Responses. Plant Cell.

[43] Franklin K.A., Lee S.H., Patel D., Kumar S.V., Spartz A.K., Gu C., Ye S., Yu P., Breen G., Cohen J.D. (2011). PHYTOCHROME-INTERACTING FACTOR 4 (PIF4) regulates auxin biosynthesis at high temperature. Proc. Natl. Acad. Sci. USA.

[44] Song X., Ma X., Li C., Hu J., Yang Q., Wang T., Wang L., Wang J., Guo D., Ge W. (2018). Comprehensive analyses of the *BES1* gene family in Brassica napus and examination of their evolutionary pattern in representative species. BMC Genom..

[45] Liu Z., Qanmber G., Lu L., Qin W., Liu J., Li J., Ma S., Yang Z., Yang Z. (2018). Genome-wide analysis of *BES1* genes in Gossypium revealed their evolutionary conserved roles in brassinosteroid signaling. Sci. China Life Sci..

[46] Feng W., Zhang H., Cao Y., Liu Y., Zhao Y., Sun F., Yang Q., Zhang X., Zhang Y., Wang Y. (2023). Maize Zm*BES1*/*BZR1*-1 transcription factor negatively regulates drought tolerance. Plant Physiol. Biochem..

[47] Gao Y., Hu J., Zhao T., Xu X., Jiang J., Li J. (2018). Genome-wide Identification and Expression Pattern Analysis of BRI1-EMS–suppressor Transcription Factors in Tomato under Abiotic Stresses. J. Am. Soc. Hortic. Sci..

[48] Song X., Li Y., Hou X. (2013). Genome-wide analysis of the AP2/ERF transcription factor superfamily in Chinese cabbage (*Brassica rapa* ssp. *pekinensis*). BMC Genom..

[49] Ma S., Ji T., Liang M., Li S., Tian Y., Gao L. (2020). Genome-Wide Identification, Structural, and Gene Expression Analysis of *BRI1-EMS-Suppressor 1* Transcription Factor Family in Cucumis sativus. Front. Genet..

[50] Xie L., Yang C., Wang X. (2011). Brassinosteroids can regulate cellulose biosynthesis by controlling the expression of CESA genes in Arabidopsis. J. Exp. Bot..

[51] Ye Q., Zhu W., Li L., Zhang S., Yin Y., Ma H., Wang X. (2010). Brassinosteroids control male fertility by regulating the expression of key genes involved in Arabidopsis anther and pollen development. Proc. Natl. Acad. Sci. USA.

[52] Lozano-Durán R., Macho A.P., Boutrot F., Segonzac C., Somssich I.E., Zipfel C. (2013). The transcriptional regulator *BZR1* mediates trade-off between plant innate immunity and growth. Elife.

[53] Li X., Liu L., Sun S., Li Y., Jia L., Ye S., Yu Y., Dossa K., Luan Y. (2022). Leaf-transcriptome profiles of phoebe bournei provide insights into temporal drought stress responses. Front. Plant Sci..

[54] Reygaert W.C. (2014). The antimicrobial possibilities of green tea. Front. Microbiol..

[55] Huang Z., Sanaeifar A., Tian Y., Liu L., Zhang D., Wang H., Ye D., Li X. (2021). Improved generalization of spectral models associated with Vis-NIR spectroscopy for determining the moisture content of different tea leaves. J. Food Eng..

[56] Zeng L., Zhou Y., Fu X., Mei X., Cheng S., Gui J., Dong F., Tang J., Ma S., Yang Z. (2017). Does oolong tea (*Camellia sinensis*) made from a combination of leaf and stem smell more aromatic than leaf-only tea? Contribution of the stem to oolong tea aroma. Food Chem..

[57] Hara Y. (2012). Elucidation of Physiological Functions of Tea Catechins and Their Practical Applications. J. Food Drug Anal..

[58] Zhang S., Takano J., Murayama N., Tominaga M., Abe T., Park I., Seol J., Ishihara A., Tanaka Y., Yajima K. (2020). Subacute Ingestion of Caffeine and Oolong Tea Increases Fat Oxidation without Affecting Energy Expenditure and Sleep Architecture: A Randomized, Placebo-Controlled, Double-Blinded Cross-Over Trial. Nutrients.

[59] Wang W., Xin H., Wang M., Ma Q., Wang L., Kaleri N.A., Wang Y., Li X. (2016). Transcriptomic Analysis Reveals the Molecular Mechanisms of Drought-Stress-Induced Decreases in Camellia sinensis Leaf Quality. Front. Plant Sci..

[60] Yu S., Li P., Zhao X., Tan M., Ahmad M.Z., Xu Y., Tadege M., Zhao J. (2021). CsTCPs regulate shoot tip development and catechin biosynthesis in tea plant (*Camellia sinensis*). Hortic. Res..

[61] Zhang Q., Shi Y., Ma L., Yi X., Ruan J. (2014). Metabolomic analysis using ultra-performance liquid chromatography-quadrupole-time of flight mass spectrometry (UPLC-Q-TOF MS) uncovers the effects of light intensity and temperature under shading treatments on the metabolites in tea. PLoS ONE.

[62] Zheng C., Ma J.Q., Ma C.L., Shen S.Y., Liu Y.F., Chen L. (2019). Regulation of Growth and Flavonoid Formation of Tea Plants (*Camellia sinensis*) by Blue and Green Light. J. Agric. Food Chem..

[63] Li Q.F., Huang L.C., Wei K., Yu J.W., Zhang C.Q., Liu Q.Q. (2017). Light involved regulation of *BZR1* stability and phosphorylation status to coordinate plant growth in Arabidopsis. Biosci. Rep..

[64] Kim B., Jeong Y.J., Corvalán C., Fujioka S., Cho S., Park T., Choe S. (2014). Darkness and gulliver2/phyB mutation decrease the abundance of phosphorylated *BZR1* to activate brassinosteroid signaling in Arabidopsis. Plant J..

[65] Roy S.W., Penny D. (2007). A very high fraction of unique intron positions in the intron-rich diatom Thalassiosira pseudonana indicates widespread intron gain. Mol. Biol. Evol..

[66] Kolodny R. (2021). Searching protein space for ancient sub-domain segments. Curr. Opin. Struct. Biol..

[67] Zhou Q., Zhao M., Xing F., Mao G., Wang Y., Dai Y., Niu M., Yuan H. (2022). Identification and Expression Analysis of CAMTA Genes in Tea Plant Reveal Their Complex Regulatory Role in Stress Responses. Front. Plant Sci..

[68] Wang Y., Tang H., Debarry J.D., Tan X., Li J., Wang X., Lee T.H., Jin H., Marler B., Guo H. (2012). MCScanX: A toolkit for detection and evolutionary analysis of gene synteny and collinearity. Nucleic Acids Res..

[69] Panchy N., Lehti-Shiu M., Shiu S.H. (2016). Evolution of Gene Duplication in Plants. Plant Physiol..

[70] Holub E.B. (2001). The arms race is ancient history in Arabidopsis, the wildflower. Nat. Rev. Genet..

[71] Xiong M., Yu J., Wang J., Gao Q., Huang L., Chen C., Zhang C., Fan X., Zhao D., Liu Q.-Q. (2022). Brassinosteroids regulate rice seed germination through the *BZR1*-RAmy3D transcriptional module. Plant Physiol..

[72] Lescot M., Déhais P., Thijs G., Marchal K., Moreau Y., Van de Peer Y., Rouzé P., Rombauts S. (2002). PlantCARE, a database of plant cis-acting regulatory elements and a portal to tools for in silico analysis of promoter sequences. Nucleic Acids Res..

[73] Gendron J.M., Wang Z.Y. (2007). Multiple mechanisms modulate brassinosteroid signaling. Curr. Opin. Plant Biol..

[74] Wang X., Niu Y., Zheng Y. (2021). Multiple Functions of MYB Transcription Factors in Abiotic Stress Responses. Int. J. Mol. Sci..

[75] Kim S.H., Lee S.H., Park T.K., Tian Y., Yu K., Lee B.H., Bai M.Y., Cho S.J., Kim T.W. (2024). Comparative analysis of *BZR1/BES1* family transcription factors in Arabidopsis. Plant J..

[76] Li M., Liu C., Hepworth S.R., Ma C., Li H., Li J., Wang S.M., Yin H. (2022). SAUR15 interaction with BRI1 activates plasma membrane H+-ATPase to promote organ development of Arabidopsis. Plant Physiol..

[77] Kojima K., Ichijo H., Naguro I. (2021). Molecular functions of ASK family in diseases caused by stress-induced inflammation and apoptosis. J. Biochem..

[78] Ohnishi T., Godza B., Watanabe B., Fujioka S., Hategan L., Ide K., Shibata K., Yokota T., Szekeres M., Mizutani M. (2012). CYP90A1/CPD, a brassinosteroid biosynthetic cytochrome P450 of Arabidopsis, catalyzes C-3 oxidation. J. Biol. Chem..

[79] Demuth J.P., Hahn M.W. (2009). The life and death of gene families. Bioessays.

[80] Oudelaar A.M., Higgs D.R. (2021). The relationship between genome structure and function. Nat. Rev. Genet..

[81] Li L., Yu X., Thompson A., Guo M., Yoshida S., Asami T., Chory J., Yin Y. (2009). Arabidopsis MYB30 is a direct target of *BES1* and cooperates with *BES1* to regulate brassinosteroid-induced gene expression. Plant J..

[82] Saito M., Kondo Y., Fukuda H. (2018). *BES1* and *BZR1* Redundantly Promote Phloem and Xylem Differentiation. Plant Cell Physiol..

[83] Roy S.W., Gilbert W. (2006). The evolution of spliceosomal introns: Patterns, puzzles and progress. Nat. Rev. Genet..

[84] Song X.M., Huang Z.N., Duan W.K., Ren J., Liu T.K., Li Y., Hou X.L. (2014). Genome-wide analysis of the bHLH transcription factor family in Chinese cabbage (*Brassica rapa* ssp. *pekinensis*). Mol. Genet. Genom..

[85] Cao X., Khaliq A., Lu S., Xie M., Ma Z., Mao J., Chen B. (2020). Genome-wide identification and characterization of the *BES1* gene family in apple (*Malus domestica*). Plant Biol..

[86] Cheng M., Yuan H., Wang R., Wang W., Zhang L., Fan F., Li S. (2023). Identification and characterization of *BES1* genes involved in grain size development of *Oryza sativa* L.. Int. J. Biol. Macromol..

[87] Cao X., Ma W., Zeng F., Cheng Y., Ma Z., Mao J., Chen B. (2023). Grape *BES1* transcription factor gene Vv*BES1*-3 confers salt tolerance in transgenic Arabidopsis. Gene.

[88] Cartharius K., Frech K., Grote K., Klocke B., Haltmeier M., Klingenhoff A., Frisch M., Bayerlein M., Werner T. (2005). MatInspector and beyond: Promoter analysis based on transcription factor binding sites. Bioinformatics.

[89] Yang C.J., Zhang C., Lu Y.N., Jin J.Q., Wang X.L. (2011). The mechanisms of brassinosteroids’ action: From signal transduction to plant development. Mol. Plant.

[90] Ibañez C., Delker C., Martinez C., Bürstenbinder K., Janitza P., Lippmann R., Ludwig W., Sun H., James G.V., Klecker M. (2018). Brassinosteroids Dominate Hormonal Regulation of Plant Thermomorphogenesis via *BZR1*. Curr. Biol..

[91] Yang M., Li C., Cai Z., Hu Y., Nolan T., Yu F., Yin Y., Xie Q., Tang G., Wang X. (2017). SINAT E3 Ligases Control the Light-Mediated Stability of the Brassinosteroid-Activated Transcription Factor *BES1* in Arabidopsis. Dev. Cell.

[92] Oh E., Zhu J.Y., Wang Z.Y. (2012). Interaction between *BZR1* and PIF4 integrates brassinosteroid and environmental responses. Nat. Cell Biol..

[93] Wang W., Lu X., Li L., Lian H., Mao Z., Xu P., Guo T., Xu F., Du S., Cao X. (2018). Photoexcited CRYPTOCHROME1 Interacts with Dephosphorylated *BES1* to Regulate Brassinosteroid Signaling and Photomorphogenesis in Arabidopsis. Plant Cell.

[94] Heijde M., Ulm R. (2012). UV-B photoreceptor-mediated signalling in plants. Trends Plant Sci..

[95] Jenkins G.I. (2014). Structure and function of the UV-B photoreceptor UVR8. Curr. Opin. Struct. Biol..

[96] Tilbrook K., Arongaus A.B., Binkert M., Heijde M., Yin R., Ulm R. (2013). The UVR8 UV-B Photoreceptor: Perception, Signaling and Response. Arab. Book.

[97] Rizzini L., Favory J.J., Cloix C., Faggionato D., O’Hara A., Kaiserli E., Baumeister R., Schäfer E., Nagy F., Jenkins G.I. (2011). Perception of UV-B by the Arabidopsis UVR8 protein. Science.

[98] Huang X., Yang P., Ouyang X., Chen L., Deng X.W. (2014). Photoactivated UVR8-COP1 module determines photomorphogenic UV-B signaling output in Arabidopsis. PLoS Genet..

[99] Liang T., Mei S., Shi C., Yang Y., Peng Y., Ma L., Wang F., Li X., Huang X., Yin Y. (2018). UVR8 Interacts with *BES1* and BIM1 to Regulate Transcription and Photomorphogenesis in Arabidopsis. Dev. Cell.

[100] Han X., Zhang J., Han S., Chong S.L., Meng G., Song M., Wang Y., Zhou S., Liu C., Lou L. (2022). The chromosome-scale genome of Phoebe bournei reveals contrasting fates of terpene synthase (TPS)-a and TPS-b subfamilies. Plant Commun..

[101] Gasteiger E., Gattiker A., Hoogland C., Ivanyi I., Appel R.D., Bairoch A. (2003). ExPASy: The proteomics server for in-depth protein knowledge and analysis. Nucleic Acids Res..

[102] Chen C., Wu Y., Li J., Wang X., Zeng Z., Xu J., Liu Y., Feng J., Chen H., He Y. (2023). TBtools-II: A “one for all, all for one“ bioinformatics platform for biological big-data mining. Molecular Plant.

[103] Cantalapiedra C.P., Hernández-Plaza A., Letunic I., Bork P., Huerta-Cepas J. (2021). eggNOG-mapper v2: Functional Annotation, Orthology Assignments, and Domain Prediction at the Metagenomic Scale. Mol. Biol. Evol..

[104] Kanehisa M., Furumichi M., Tanabe M., Sato Y., Morishima K. (2017). KEGG: New perspectives on genomes, pathways, diseases and drugs. Nucleic Acids Res..

[105] Otasek D., Morris J.H., Bouças J., Pico A.R., Demchak B. (2019). Cytoscape Automation: Empowering workflow-based network analysis. Genome Biol..

[106] Winters M., Eskes M., Weir A., Moen M.H., Backx F.J., Bakker E.W. (2013). Treatment of medial tibial stress syndrome: A systematic review. Sports Med..

[107] Day R.W., Quinn G.P. (1989). Comparisons of Treatments After an Analysis of Variance in Ecology. Ecol. Monogr..

